# Transfusion increased skin blood flow when initially low in volume-resuscitated patients without acute bleeding

**DOI:** 10.3389/fmed.2023.1218462

**Published:** 2023-10-04

**Authors:** Elaine Cavalcante dos Santos, Péter Bakos, Diego Orbegozo, Jacques Creteur, Jean-Louis Vincent, Fabio Silvio Taccone

**Affiliations:** Department of Intensive Care Medecine, Erasme University Hospital, Université Libre de Bruxelles (ULB), Brussels, Belgium

**Keywords:** tissue perfusion, anemia, red blood cell transfusion, skin blood flow, skin laser doppler, non-bleeding critical patients, sepsis

## Abstract

**Background:**

Alterations in skin blood flow is a marker of inadequate tissue perfusion in critically ill patients after initial resuscitation. The effects of red blood cell transfusions (RBCT) on skin perfusion are not described in this setting. We evaluated the effects of red blood cell transfusions on skin tissue perfusion in critically ill patients without acute bleeding after initial resuscitation.

**Methods:**

A prospective observational study included 175 non-bleeding adult patients after fluid resuscitation requiring red blood cell transfusions. Using laser Doppler, we measured finger skin blood flow (SBF) at skin basal temperature (SBF_BT_), together with mean arterial pressure (MAP), heart rate (HR), hemoglobin (Hb), central venous pressure (CVP), lactate, and central or mixed venous oxygen saturation before and 1 h after RBCT. SBF responders were those with a 20% increase in SBF_BT_ after RBCT.

**Results:**

Overall, SBF_BT_ did not significantly change after RBCT [from 79.8 (4.3–479.4) to 83.4 (4.9–561.6); *p* = 0.67]. A relative increase equal to or more than 20% in SBF_BT_ after RBCT (SBF responders) was observed in 77/175 of RBCT (44%). SBF responders had significantly lower SBF_BT_ [41.3 (4.3–279.3) vs. 136.3 (6.5–479.4) perfusion units; *p* < 0.01], mixed or central venous oxygen saturation (62.5 ± 9.2 vs. 67.3% ± 12.0%; *p* < 0.01) and CVP (8.3 ± 5.1 vs. 10.3 ± 5.6 mmHg; *p* = 0.03) at baseline than non-responders. SBF_BT_ increased in responders [from 41.3 (4.3–279.3) to 93.1 (9.8–561.6) perfusion units; *p* < 0.01], and decreased in the non-responders [from 136.3 (6.5–479.4) to 80.0 (4.9–540.8) perfusion units; *p* < 0.01] after RBCT. Pre-transfusion SBF_BT_ was independently associated with a 20% increase in SBF_BT_ after RBCT. Baseline SBF_BT_ had an area under receiver operator characteristic of 0.73 (95% CI, 0.68–0.83) to predict SBF_BT_ increase; A SBF_BT_ of 73.0 perfusion units (PU) had a sensitivity of 71.4% and a specificity of 70.4% to predict SBF_BT_ increase after RBCT. No significant differences in SBF_BT_ were observed after RBCT in different subgroup analyses.

**Conclusion:**

The skin blood flow is globally unaltered by red blood cell transfusions in non-bleeding critically ill patients after initial resuscitation. However, a lower SBF_BT_ at baseline was associated with a relative increase in skin tissue perfusion after RBCT.

## Introduction

Anemia is a prevalent issue in critically ill patients (1, 2). Despite a decline in red blood cell transfusions (RBCT) usage over time (3), they remain commonly administered to intensive care unit (ICU) patients (4), even in the absence of active bleeding (5). It is important to note that RBCT may have either beneficial effects, such as increasing global oxygen delivery and improving tissue oxygenation, or harmful effects, including transfusion-related acute lung injury (TRALI), transfusion-associated circulatory overload (TACO), and immunosuppression with increased risk of infections (6–10). Consequently, deciding whether to transfuse an ICU patient is a significant challenge for clinicians and should be personalized based on specific triggers.

Current guidelines recommend a restrictive RBCT strategy with a hemoglobin (Hb) threshold of 7 g/dL for considering transfusions in non-bleeding ICU patients (11). However, a fixed Hb value does not adequately reflect tissue perfusion or oxygen requirements in this setting (12, 13). Additionally, hematocrit is lower in capillaries than in large vessels (arteries and veins), making systemic Hb an inadequate parameter for microvascular oxygen content and viscosity (14–16). Furthermore, systemic endpoints for estimating tissue perfusion and oxygenation to guide RBCT have yielded conflicting results (17–20). A recent meta-analysis demonstrated that RBCT significantly increased oxygen delivery and consumption, while reducing the oxygen extraction ratio, without affecting cardiac output, in critically ill patients (21). These effects were particularly evident in septic patients, with a wide range of RBCT effects among other subgroups (21). It is important to note that microvascular alterations and tissue hypoxia may persist in the presence of normal values for systemic variables, including blood pressure, cardiac output, lactate levels, or oxygen delivery (22–27). Consequently, RBCT administration should not solely rely on these systemic variables but should be based on tissue perfusion and/or oxygenation.

Some studies have indicated that RBCT have heterogeneous effects on sublingual microcirculation, with either improved or altered microvascular density, in critically ill patients (12, 13). Similar results were observed when measuring muscular oxygenation saturation and applying a vascular occlusion test (VOT), with those patients with impaired baseline microvascular reactivity showing an improvement while the others showed a deterioration of microvascular function after RBCT (28). A recent study suggested that low baseline brain oxygen pressure values could predict a significant improvement in brain oxygenation after RBCT in critically ill patients with acute brain injury (29). However, these methods have some limitations. Tissue oxygenation with VOT requires a dedicated probe and software that are no longer available, and repeated measurements could affect baseline results, limiting its routine applicability at the bedside (30–32). Sublingual microcirculation assessment using videomicroscopy can be challenging in awake and/or agitated patients. Brain oxygen pressure monitoring is expensive and invasive and is only feasible in brain-injured patients (33). In this context, Skin Laser Doppler (SLD) is a non-invasive and straightforward technique for directly assessing skin blood flow (SBF) and tissue perfusion (34, 35). This technique uses an optical fiber to direct light from a low-power laser source to the skin and to collect the back-scattered light; the shift in light wavelength is proportional to the red blood cell velocity in the studied skin area, providing a non-invasive measurement of SBF (35, 36). However, no studies have evaluated the effects of RBCT on tissue perfusion using this technology in non-bleeding ICU patients after resuscitation.

The aim of this study was therefore to evaluate the effects of RBCT on SBF and to identify predictors of skin blood flow response to RBCT in non-bleeding critically ill patients after initial resuscitation.

## Methods

### Study population

This prospective observational study was conducted at the Department of Intensive Care at Erasme Hospital (Belgium) from September 2018 to April 2021. The Ethics Committee approved the study (protocol number P2018/588), and all patients and/or their next of kin provided informed consent.

Consecutive adult patients (>18 years of age) admitted to the 34-bed ICU during the study period who did not have evidence of active bleeding and who were considered euvolemic and for whom the attending physicians decided to administer RBCT were eligible for inclusion. Exclusion criteria were pregnancy, RBCT transfusion within the preceding 24 h, the presence of any skin lesion that prevented the use of SLD, and mechanical assist devices or renal support.

### Transfusion policy

The decision to transfuse the patient was made by the responsible physician following a local transfusion policy that considers a hemoglobin level below 7–8 g/dL as an indication for transfusion. Packed red blood cells (RBCs) units were obtained from the blood bank at the Erasme Hospital Belgian Red Cross and were transfused in the ICU. All RBC units underwent leukocyte reduction through filtration before storage. A storage solution consisting of saline-adenine-glucose-mannitol was added to the RBCs before storage, and the maximum allowed storage period was 42 days.

### Data collection

Demographic data, disease severity, and the main diagnosis were collected from all patients. The Acute Physiology and Chronic Health Evaluation II (APACHE II) score (37) was calculated using the worst data within the first 24 h of ICU admission, and the Sequential Organ Failure Assessment (SOFA) score (38) was calculated upon admission, on the day of inclusion in the study, and the following day. In addition, mean arterial pressure (MAP), heart rate (HR), central venous pressure (CVP), central or mixed venous oxygen saturation (ScvO_2_ or SvO_2_), arterial lactate levels, arterial and venous blood gas analyses (ABL700, Radiometer, Copenhagen, Denmark), body temperature (T, °C), hematocrit (Htc) and Hb were collected for all patients before RBCT. All measurements, including SBF, were repeated at the end of RBCT. The survival status was recorded 28 days after RBCT. The use of vasopressors, sedatives, or mechanical ventilation on the study day was also documented. If two units were transfused on the same day, data were collected and measurements were obtained only after the first transfusion. Hemodynamic, Hb, and SBF variables were measured immediately before and 1 h after RBCT. During the measurements, the patient’s position was kept constant. Measurements were classified as “interference” when the dose of vasopressor/inotropic and sedative/analgesic agents was not maintained at a constant level over time, as determined by the responsible physician.

### Skin blood flow measurements

SBF was measured using the SLD device Periflux System 5000 (Perimed, Jarfalla, Sweden, RRID: SCR_015962) at the same time points. The thermostatic probe was positioned on the tip of the index finger, and the SBF at skin basal temperature (SBF_BT_) was recorded for 3 min. Subsequently, the skin probe temperature was then immediately increased to 37°C, and the SBF (SBF_37_) was again recorded after 3 min. The thermal challenge test (TCT) at a skin temperature of 37°C was calculated using the following formula: SBF_37_–SBF_BT_/37-BT (arbitrary units, PU/C). The measurements were obtained using the same device in all patients, and the skin probe was placed on the same studied area during all SBF measurements. Patients were instructed not to move their hands or change the bed’s position during SBF measurements and the transfusion period.

### Definitions

Skin blood flow “responders” to RBCT were defined as patients showing a relative increase in SBF_BT_ of at least 20% from baseline values. This threshold was arbitrarily chosen and selected to identify a change in tissue perfusion that would be clinically significant. The relative change in SBF_BT_ after RBCT was calculated using the formula: ((SBF_BT_ after RBCT – SBF_BT_ at baseline)/SBF_BT_ baseline) × 100%. RBCT was considered “early” if administered within 3 days of ICU admission. Sepsis was diagnosed according to standard criteria. Acute neurological disease (AND) was defined as patients admitted with one of the following conditions: acute brain injury (subarachnoid hemorrhage, traumatic brain injury, and intracerebral hemorrhage), acute ischemic stroke, or meningoencephalitis. Chronic anemia was defined based on a known history of anemia/or decreased hematocrit (Hct) and Hb concentration on previous laboratory tests (39). Fluid administration before study day was calculated by dividing the total fluid balance (from admission to the day of RBC transfusion) by the number of days to have a daily fluid balance reported in liters per day (L/day). Lastly, “SOFA responder” was defined as patients showing a reduction of the SOFA score by ≥1 point within 24 h after RBCT compared to the baseline SOFA score.

### Study outcomes

The primary outcome of this study was the proportion of patients who showed a tissue response of SBF following the first RBCT. The secondary outcome included identifying variables that could predict a tissue response to RBCT and subgroup analyses of tissue response based on the presence of sepsis or the timing of transfusion. For both primary and secondary outcomes, the analysis included the first transfusion enrolled in the study. A sensitivity analysis of the primary outcome was performed, including all RBCT in patients who received multiple transfusions during their ICU stay.

### Sample size calculation

Based on the hypothesis of a relative increase greater than or equal to 20% in SBF_BT_ (Baseline SBF_BT_ = 115.0 ± 100.0 vs. SBF_BT_ after RBCT = 138.0 ± 100.0), we estimated that a sample size of 95 patients was needed to have a power of 80% with an alpha error of 5% to test the study hypothesis.

### Statistical analysis

Normality distribution was evaluated using the Kolmogorov–Smirnov test for each parameter. Data are presented as median (25th–75th percentiles) or mean with standard deviation, as appropriate. Categorical variables were described and counted (%) and differences between groups were assessed using the chi-square test or Fisher’s exact test as required. A Wilcoxon’s test or Paired Student’s tests were used to compare the pre-transfusion and post-transfusion values, as appropriate. Mann–Whitney *U*-test or Unpaired Student’s tests were used for data with non-normal and normal distribution, respectively. Multivariable logistic regression models were performed to investigate predictors of tissue response to RBCT, which included as covariates in the model all variables with *value of p* <0.2 at univariate analysis. A generalized mixed model (GMM) with logit link and covariance matrix with the first-order autoregressive structure was used to identify baseline variables that were independently associated with a significant SBF_BT_ increase of all included RBCT (sensitivity analysis); variables with a *value of p* less than 0.2 in the univariate analysis were included. The odds ratio with their 95% CI was computed for all variables. The independence of errors and the presence of collinearity were also checked. The area under the receiving operator curve (AUROC) was computed to assess the predictive value of pre-transfusion SBF_BT_ for tissue response to RBCT; the optimal predictive cut-off score was determined using Youden’s index. All considered tests were two-tailed and a *value of p* <0.05 was considered to be statistically significant. Statistical analyses were performed using SPSS Statistics for Windows, version 27.0 (IBM, Chicago, United States).

## Results

### Study population

During the study period, 195 non-bleeding patients received 252 RBCT and were screened. Twenty-six RBCT from 20 patients were excluded because of severe agitation (*n* = 6), refused consent (*n* = 2), start ultrafiltration at the moment of RBCT (*n* = 5), concomitant fluid challenge (*n* = 3), interruption of transfusion due to adverse effects (n = 1), other procedures (endotracheal intubation and tracheostomy; *n* = 2), need for imaging tests (*n* = 3), isolation because of multi-resistant bacteria (*n* = 1), end-of-life procedure (*n* = 1), acute bleeding (*n* = 1), and skin lesions (*n* = 1). As such, 175 patients receiving 175 RBCT were included in the final analysis for the primary and secondary outcomes. Also, 175 patients receiving 226 RBCT were included in the sensitivity analysis of the primary outcome (Supplementary Figure 1).

The characteristics of the study cohort (n = 175; mean age 63.0 ± 15.0 years; 63.4% male) are shown in Table 1; 89 (51.0%) patients had medical admissions, in particular sepsis (*n* = 48, 27.4%). RBCT were given after a median of 4 (0–42) days after ICU admission; 6 (3.4%) RBCT had interference. Twenty-nine patients (16.6%) died 28 days after the study inclusion.

**Table 1:** Characteristics of the study population, according to the tissue response to transfusion (SBF responders vs. SBF non-responders) in the first RBCT (*n* = 175).

	All Patients (*n* = 175)	SBF responders (*n* = 77)	SBF non-responders (*n* = 98)	Value of *p*
Age, years	63.0 ± 15.0	66.0 ± 14.0	59.0 ± 14.4	<0.01
Male Gender, *n* (%)	111.0(63.4)	48.0(62.3)	63.0(64.3)	0.79
APACHE II score on admission	22.0 ± 7.2	22.0 ± 7.4	21.0 ± 7.1	0.66
SOFA score on admission	9.4 ± 3.0	9.4 ± 2.8	9.5 ± 3.2	0.80
Hemoglobin on admission, g/dL	10.0 ± 2.3	9.9 ± 2.2	10.2 ± 2.4	0.34
Hematocrit on admission (%)	30.1 ± 6.9	29.7 ± 6.5	30.4 ± 7.2	0.50
**Admission type, *n* (%)**				
Surgical	72.0 (41.1)	32.0(41.6)	40.0(40.8)	0.87
Medical	89.0 (51.0)	38.0(49.4)	51.0(52.0)	
Trauma	14.0 (8.0)	7.0(9.1)	7.0(7.1)	
**Comorbidities, *n* (%)**				
Arterial hypertension	81.0 (46.3)	35.0(45.5)	46.0(46.9)	0.85
Diabetes	41.0 (23.4)	17.0(22.1)	24.0(24.5)	0.71
Coronary arterial disease	44.0 (25.1)	20.0(26.0)	24.0(24.5)	0.82
Chronic renal disease	42.0 (24.0)	21.0(27.3)	21.0(21.4)	0.37
COPD	20.0 (11.4)	7.0(9.1)	13.0(13.3)	0.39
Liver cirrhosis	21.0 (12.0)	8.0(10.4)	13.0(13.3)	0.56
Peripheral artery disease	22.0 (12.6)	12.0(15.6)	10.0(10.2)	0.29
History of smoking	26.0 (14.9)	11.0(17.3)	15.0(15.3)	0.85
Immunosuppression	43.0 (24.6)	20.0(26.0)	23.0(23.5)	0.70
No-metastatic solid cancer	17.0 (9.7)	90 (11.7)	8.0(8.2)	0.43
Metastatic solid cancer	4.0 (2.3)	2.0(2.6)	2.0(2.0)	0.81
Hematological cancer	10.0 (5.7)	4.0(5.2)	6.0(6.1)	0.99
Chronic anemia	64.0 (36.6)	29.0(37.7)	35.0(35.7)	0.79
**Reasons for admission, *n* (%)**				
Sepsis/septic shock	48.0 (27.4)	24.0(31.2)	24.0(24.5)	<0.01
Respiratory failure	8.0 (4.6)	6.0(7.8)	2.0(2.0)	
Hypovolemic shock	26.0 (14.9)	15.0(19.5)	11.0(11.2)	
Cardiogenic shock	31.0 (17.7)	11.0(14.3)	20.0(20.4)	
Trauma	5.0 (2.9)	3.0(3.9)	2.0(2.0)	
Acute neurological disease	40.0(22.9)	8.0(10.4)	32.0(32.7)	
Others	17.0 (9.7)	10.0(13.0)	7.0(7.1)	
**RBCT characteristics**				
Volume, mL	266.3 ± 17.0	267.0 **±** 19.4	266.6 **±** 15.1	0.88
Age, days	23.1 ± 8.0	23.7 **±** 8.7	22.6 **±** 7.4	0.36
**Characteristics of measurements**				
Time in ICU before study inclusion (days)	4.0 (0–42.0)	4.0(0–42.0)	4.0(0–35.0)	0.32
Interference, *n* (%)	6.0 (3.4)	2.0(2.6)	4.0(4.1)	0.59
Fluid administration before study inclusion (L/day)	0.95 (−5.00–9.50)	0.94 (−5.00–9.50)	0.97 (−1.30–9.40)	0.69
**On the day of the first RBCT, *n* (%)**				
Mechanical ventilation	79.0 (45.1)	27.0(35.1)	52.0(53.1)	0.02
Sedation	45.0 (25.7)	13.0(16.9)	32.0(32.7)	0.02
Vasopressors	111 (63.4)	45.0(58.4)	66.0(67.3)	0.27
**During ICU stay, *n* (%)**				
28-day mortality	29.0 (16.6)	11.0(14.3)	18.0(18.4)	0.47
SOFA responder	103.0 (58.9)	51.0(66.2)	52.0(53.1)	0.08

Data are reported as mean (SD), median (IQRs), or count (%). APACHE II, Acute Physiologic Assessment and Chronic Health Evaluation Scoring System II; SOFA, Sequential Organ Failure Assessment; COPD, Chronic Obstructive Pulmonary Disease; ICU, Intensive Care Unit.

### Skin blood flow response to RBCT

**Figure 1:**
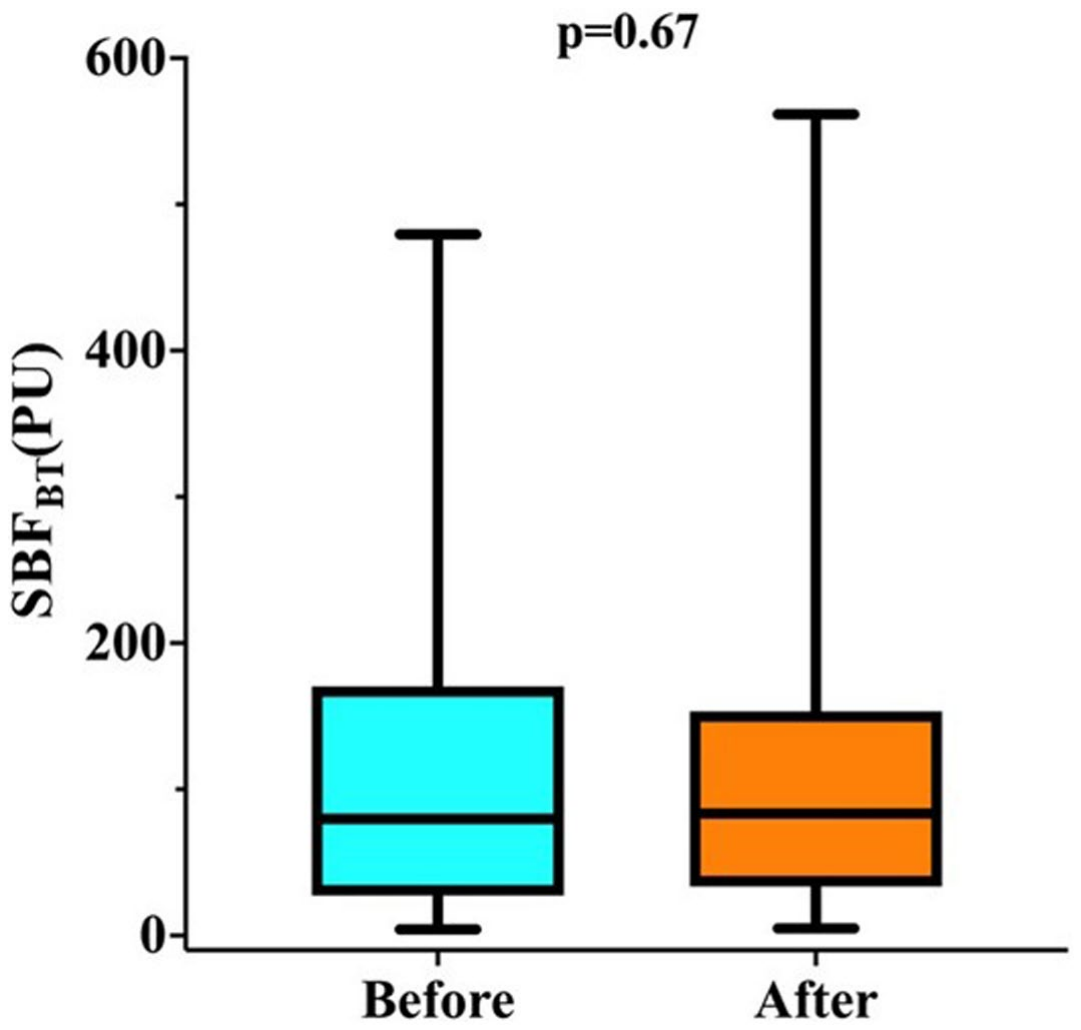
Changes in skin blood flow at basal temperature (SBF_BT_) before and after RBCT in all 175 patients.

The mean Hb before RBCT was 7.5 ± 0.7 g/dL and the mean SOFA score on the day of RBCT was 8.5 ± 3.1 (Table 2); median pre-transfusion SBF_BT_ was 79.8 (4.3–479.4) PU (Table 2). There were no significant changes in absolute SBF_BT_ values in the whole population after RBCT (Figure 1; Table 2); RBCT resulted in a significant increase in Hb (from 7.5 ± 0.7 to 8.7 ± 0.8 g/dL; *p* < 0.01), Htc (from 19.1 ± 2.8 to 23.0 ± 3.4%; *p* < 0.01), mean arterial pressure (from 80.0 ± 13.3 to 82.0 ± 13.6 mmHg; *p* < 0.01) and venous oxygen saturation (from 65.2 ± 11.1 to 67.7 ± 9.9%; *p* < 0.01; Table 2).

**Table 2:** Pre- and post-transfusion changes in collected variables for the first RBCT (*n* = 175).

	Baseline	Day-1	Value of *p*
SOFA score	8.5 ± 3.1	7.5 ± 3.5	<0.01
RDW, %	16.0 (12.0–28.0)	16.0 (12.0–29.0)	<0.01

	Baseline	1-h	Value of *p*
Hemoglobin, g/dL	7.5 ± 0.7	8.7 ± 0.8	<0.01
Hematocrit, %	19.1 ± 2.8	23.0 ± 3.4	<0.01
Body temperature, °C	37.0 ± 0.8	37.0 ± 0.8	0.56
Mean arterial pressure, mmHg	80.0 ± 13.3	82.0 ± 13.6	<0.01
Heart rate, bpm	92.0 ± 17.1	91.0 ± 16.6	0.24
Central venous pressure, mmHg	9.4 ± 5.5	9.2 ± 5.4	0.20
Noradrenaline dose, mcg/kg/min	0.15(0.00–4.00)	0.13(0.00–3.00)	0.11
Lactate concentration, mmol/L	1.2(0.3–5.6)	1.2(0.3–5.4)	0.80
Arterial oxygen saturation, %	98.0(85.0–100.0)	98.1(86.0–100.0)	0.61
Venous oxygen saturation, %	65.2 ± 11.1	67.7 ± 9.9	<0.01
Urinary output (mL/Kg/h)	1.4(0.1–30.0)	2.5(0.1–35.3)	<0.01
Finger Temperature baseline, °C	30.4 ± 3.1	30.3 ± 3.2	0.43
SBF_BT_, PU	79.8 (4.3–479.4)	83.4(4.9–561.6)	0.67
SBF_37_, PU	109.7 (7.7–493.2)	120.6 (13.6–585.3)	0.59
∆SBF/∆T, PU/°C	2.7 (−21.9–32.9)	2.0 (−57.1–48.2)	0.84

Data are reported as mean (SD), or median (IQRs). SOFA, Sequential Organ Failure Assessment; RDW, red blood cell distribution width; SBF_BT_, skin blood flow at basal temperature; SBF_37_, skin blood flow at 37°C; ∆SBF/∆T = (SBF_37_–SBF_BT_/T37°C-T at baseline); PU, perfusion units.

### Predictors of skin blood flow response to RBCT

**Figure 2:**
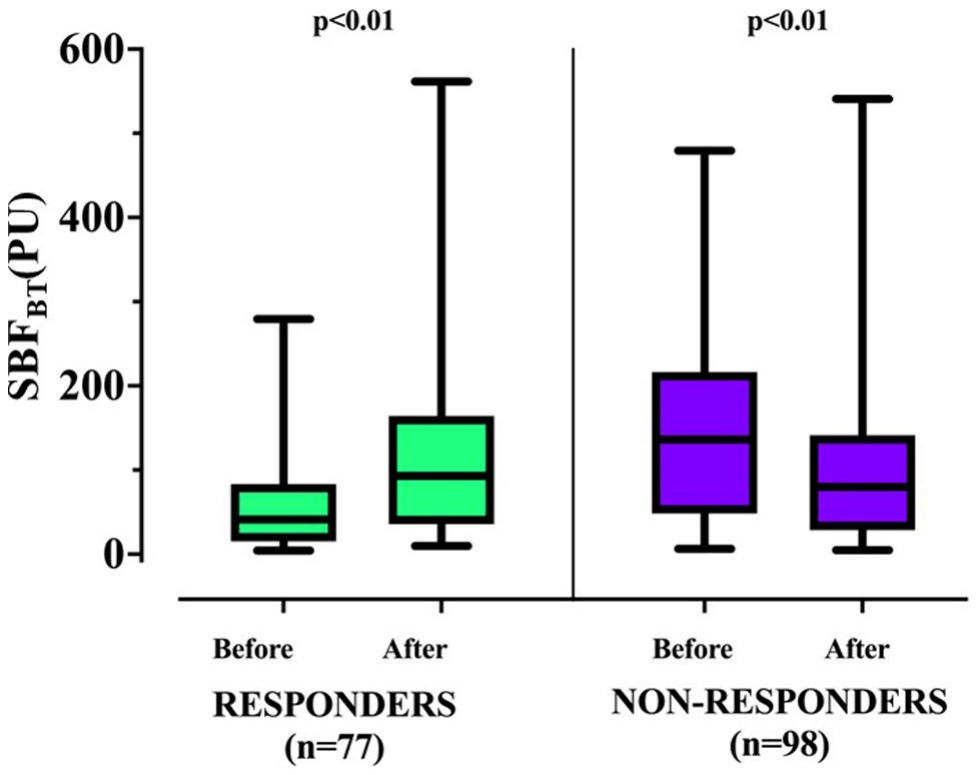
Changes in skin blood flow at basal temperature (SBF_BT_) before and after RBCT according to the response in tissue perfusion. *p-*values indicate differences between before and after RBCT in each subgroup.

A total of 77/175 (44.0%) patients had their first RBCT resulting in a significant increase in SBF_BT_ after RBCT (responders). The clinical characteristics of SBF responders and non-responders are shown in Table 1. SBF responders were older, had a less frequent acute neurological disease, and received less frequent sedatives and mechanical ventilation than non-responders. There was no significant difference in the Hb on admission, Htc on admission, frequency of chronic anemia, or median fluid administration before study inclusion between groups (Table 1). There was no significant difference in the pre-transfusion Hb, Htc, HR, MAP, noradrenaline dose, or arterial lactate concentrations between groups (Table 3); CVP, venous oxygen saturation, and SBF_BT_ were significantly lower in SBF responders than in non-responders (Table 3). Also, baseline SBF_BT_ showed a significant increase after RBCT in SBF responders (from 41.3 [4.3–279.3] to 93.1[9.8–561.6] PU; *p* < 0.01), whereas it significantly decreased in non-responders (from 136.3[6.5–479.4] to 80.0[4.9–540.8] PU; *p* < 0.01; Figure 2; Table 3).

**Table 3:** Comparison between pre-and post-transfusion variables, according to tissue response.

SBF responders (*n* = 77)			
	Baseline	Day-1	Value of *p*
SOFA score	8.4 ± 2.9	7.1 ± 3.3	<0.01
RDW, %	16.0(13.0–28.0)	16.0(13.0–29.0)	<0.01

	Baseline	1-h	
Hemoglobin, g/dL	7.5 ± 0.7	8.8 ± 0.8	<0.01
Hematocrit, %	19.5 ± 2.7	23.3 ± 3.3	<0.01
Body temperature, °C	37.0 ± 0.7	37.0 ± 0.7	0.41
Mean arterial pressure, mmHg	78.3 ± 11.7	79.0 ± 12.5	0.69
Heart rate, bpm	92.2 ± 15.0	89.0 ± 13.6	0.02
Central venous pressure, mmHg	8.3 ± 5.1	8.0 ± 5.1	0.90
Noradrenaline dose, mcg/kg/min	0.15 (0.01–0.90)	0.13(0.0–0.90)	0.37
Lactate concentration, mmol/L	1.1 (0.3–5.1)	1.2(0.3–5.2)	0.82
Arterial oxygen saturation, %	98.0 (89.8–100)	98.0 (86.0–100.0)	0.51
Venous oxygen saturation, %	62.5 ± 9.2	66.1 ± 9.0	<0.01
Urinary output (mL/Kg/h)	1.1(0.1–8.0)	2.4(0.1–10.3)	<0.01
Finger temperature baseline, °C	29.4 ± 3.1	30.0 ± 3.2	0.05
SBF_BT_, PU	41.3 (4.3–279.3)	93.1(9.8–561.6)	<0.01
SBF_37_, PU	90.4 ± 64.2	145.2 ± 585.3	<0.01
∆SBF/∆T, PU/°C	2.7(−1.4–22.6)	2.3(−17.3–48.2)	0.68

SBF non-responders (*n* = 98)			
	Baseline	Day-1	Value of *p*
SOFA score	8.6 ± 3.3	7.8 ± 3.6	<0.01
RDW, %	16.0(12.0–26.0)	16.0(12.0–25.0)	0.38

	Baseline	1-h	Value of *p*
Hemoglobin, g/dL	7.4 ± 0.7	8.6 ± 0.9	<0.01
Hematocrit, %	18.8 ± 2.8	22.7 ± 3.5	<0.01
Body temperature, °C	37.0 ± 0.9	37.0 ± 0.8	0.87
Mean arterial pressure, mmHg	81.3 ± 14.4	85.0 ± 13.9	<0.01
Heart rate, bpm	91.0 ± 18.7	92.0 ± 18.6	0.64
Central venous pressure, mmHg	10.3 ± 5.6	10.0 ± 5.6	0.13
Noradrenaline dose, mcg/kg/min	0.15(0.0–4.0)	0.14(0.0–3.0)	0.20
Lactate concentration, mmol/L	1.2(0.4–5.6)	1.3(0.5–5.4)	0.92
Arterial oxygen saturation, %	98.3(85.0–100)	98.0(92.0–100.0)	0.21
Venous oxygen saturation, %	67.3 ± 12.0	68.9 ± 10.6	0.08
Urinary output (mL/Kg/h)	1.5(0.1–30.0)	2.6(0.1–35.3)	<0.01
Finger temperature baseline, °C	31.2 ± 3.0	30.6 ± 3.1	<0.01
SBF_BT_, PU	136.3(6.5–479.4)	80.0(4.9–540.8)	<0.01
SBF_37_, PU	166.8 ± 102.7	124.0(13.6–424.0)	<0.01
∆SBF/∆T, PU/°C	2.6(−21.9–32.9)	2.6(−21.9–32.9)	0.98

Baseline values			
	Responders (*n* = 77)	Non-responders (*n* = 98)	Value of *p*
SOFA score on study day	8.4 ± 2.9	8.6 ± 3.3	0.67
Hemoglobin, g/dL	7.5 ± 0.7	7.4 ± 0.7	0.20
Hematocrit, %	19.5 ± 2.7	18.8 ± 2.8	0.10
RDW, %	16.0(13.0–28.0)	16.0(12.0–26.0)	0.86
Body temperature (°C)	37.0 ± 0.7	37.0 ± 0.9	0.42
Mean arterial pressure, mmHg	78.3 ± 11.7	81.3 ± 14.4	0.15
Heart rate, bpm	92.2 ± 15.0	91.0 ± 18.7	0.65
Central venous pressure, mmHg	8.3 ± 5.1	10.3 ± 5.6	0.03
Noradrenaline dose, mcg/kg/min	0.15 (0.01–0.90)	0.15(0.0–4.0)	0.55
Lactate concentration, mmol/L	1.1 (0.3–5.1)	1.2(0.4–5.6)	0.92
Arterial oxygen saturation (%)	98.0 (89.8–100)	98.3(85.0–100)	0.23
Venous oxygen saturation, %	62.5 ± 9.2	67.3 ± 12.0	<0.01
Urinary output (mL/Kg/h)	1.1(0.1–8.0)	1.5(0.1–30.0)	<0.01
Finger temperature baseline, °C	29.4 ± 3.1	31.2 ± 3.0	<0.01
SBF_BT_, PU	41.3 (4.3–279.3)	136.3(6.5–479.4)	<0.01
SBF_37_, PU	90.4 ± 64.2	166.8 ± 102.7	<0.01
∆SBF/∆T, PU/°C	2.7(−1.4–22.6)	2.6(−21.9–32.9)	0.57

Data are reported as mean (SD), or median (IQRs). SOFA, Sequential Organ Failure Assessment; RDW, red blood cell distribution width; SBF_BT,_ skin blood flow at basal temperature; SBF_37_, skin blood flow at 37°C; ∆SBF/∆T = (SBF_37_–SBF_BT_/T37°C-T at baseline); PU, perfusion units.

In the multivariable analysis, lower baseline SBF_BT_, older age, and the absence of an acute neurological disease were independently associated with SBF response to RBCT (Supplementary Table 1). The AUROC curve for baseline SBF_BT_ to predict skin blood flow response to RBCT was 0.76 (95% CI, 0.68–0.83; *p* < 0.001). The optimal baseline SBF_BT_ to predict skin blood flow response to RBCT was 73.0 PU, with 71.4% sensibility and 70.4% specificity (Supplementary Figure 2). In the sensitivity GMM analysis (*n* = 226 RBCT), lower baseline SBF_BT_, older age, and the absence of an acute neurological disease were independently associated with skin blood flow response to RBCT (Supplementary Table 2).

### Subgroup analysis

Characteristics of patients according to the timing of RBCT or underlying disease are shown in Supplementary Tables S3–S6. Sixty-two out of 175 (36.0%) patients received early RBCT (Supplementary Tables S3, S4); there was no difference between the proportion of RBCT being associated with a significant increase in SBF_BT_ (31/62, 50.0% vs. 46/113, 40.7%, *p* = 0.24) between the early and the late groups (Supplementary Table S3). There were no significant differences in baseline SBF_BT_ (Supplementary Table S3) and no significant differences in changes in SBF_BT_ after RBCT was observed between groups (Supplementary Table S4; Supplementary Figure 3).

Sixty-three out of 175 (36%) patients had sepsis (Supplementary Table 5); there was no difference between the proportion of RBCT being associated with a significant increase in SBF_BT_ (25/63, 39.7% vs. 52/112, 46.4%, *p* = 0.39) between groups (Supplementary Table 5). There were no significant differences in baseline SBF_BT_ (Supplementary Table 5) and no significant differences in changes in SBF_BT_ after RBCT was observed between groups (Supplementary Table 6; Supplementary Figure 4).

## Discussion

In this prospective study, we found that RBCT did not consistently affect skin tissue perfusion, as assessed using laser Doppler, in critically ill patients after resuscitation without acute bleeding. However, there was substantial interindividual variability in the response to RBCT; 44% of patients showed a significant increase in SBF_BT_. Patients who responded to RBCT had lower SBF_BT_ values at baseline than non-responders, and the relative increase in tissue perfusion after RBCT was higher when baseline SBF_BT_ was lower. We found no relationship between skin tissue perfusion response to RBCT and storage time, the presence of sepsis, or systemic hemodynamics.

The ultimate goal of RBCT is to improve tissue oxygenation (12). However, RBCT is still often given based on a specific Hb trigger, despite Hb being a poor surrogate for estimating oxygen delivery (DO_2_), tissue perfusion, oxygenation, or cellular metabolic needs (12, 40, 41). Thus, monitoring tissue perfusion, oxygenation, and microcirculation could be a useful tool for identifying patients who require interventions to improve microvascular flow or oxygenation, with RBCT being one possible intervention (15). Previous studies have reported heterogeneous effects of RBCT on microcirculatory parameters. In septic and trauma patients, RBCT did not affect sublingual microcirculation assessed by videomicroscopy, despite increases in Hb, MAP, and global DO_2_ (12, 42). However, improved microcirculation was observed in patients with altered perfused capillary density at baseline, while the opposite was reported in those with normal microvascular flow. In septic patients, RBCT did not affect muscle oxygen saturation, oxygen consumption, or microvascular reactivity measured using near-infrared spectroscopy (NIRS) (43). However, changes in tissue oxygenation and oxygen consumption were negatively correlated with baseline values, suggesting a larger response to RBCT in patients with impaired tissue oxygenation at baseline (28). Other studies, including mixed ICU populations, trauma, hemorrhagic shock, or ECMO patients, reported that RBCT directly improved microcirculation assessed by videomicroscopy (13, 42, 44–46). While all studies reported that the effects of RBCT on microcirculation were independent of hemodynamic parameters, our study highlights how skin tissue perfusion can variably respond to RBCT and suggests that discrepancies among studies may be due to differences in patient selection and the techniques used.

Undoubtedly, RBCT cannot be considered the optimal intervention for consistently improving tissue oxygenation and perfusion. In critical illness, after initial resuscitation, some patients may have a normal microvascular function and should not receive RBCT based solely on hemoglobin values, as this could potentially be harmful, as demonstrated in our study and other studies. However, RBCT may provide beneficial effects on microvascular density, tissue perfusion, tissue oxygenation, and oxygen consumption when microvascular function is impaired, and systemic hemodynamics are restored within accepted therapeutic ranges. Our study found that 44% of patients had skin blood flow responders to RBCT. Therefore, it is essential to identify predictors of SBF response to RBCT to individualize transfusion policies in clinical practice. Specifically, lower baseline values of skin blood flow basal tone could predict skin tissue response to RBCT with moderate accuracy. These results are consistent with previous reports, which suggest low baseline functional capillary density and tissue oxygenation as predictors of RBCT response (12, 28). Although we did not concurrently assess sublingual microcirculation with videomicroscopy or tissue oxygenation with NIRS, assessing tissue perfusion using SBF has some potential advantages. First, the technique is easy to use, even in non-intubated and agitated patients, who are frequently difficult to assess with videomicroscopy (27, 34). Second, SBF requires a very short learning phase and provides accurate inter-operator reproducibility during pilot studies in our service (data not shown). Third, altered SBF has been associated with the severity of the underlying disease, has important prognostic value, and responds to other clinically relevant interventions in ICU patients (27, 47, 48).

The interindividual variability of tissue response after RBCT could be related to other factors. The presence of an underlying neurological disease was an independent predictor of non-response to RBCT. Microvascular skin flow is controlled by the autonomic nervous system (ANS) (49), and arterio-venous shunts provide a low-resistance pathway by which blood flow can bypass capillaries and thus be drained from the arterioles to venules. The shunts are maintained in a constricted state by a sympathetic tone (50). The neurological disease causes loss of this tone and then the shunts open diverting blood flow from the skin (51). The lack of significant changes in tissue perfusion in neurological patients might be related to ANS dysfunction. Higher age was also an independent predictor of skin tissue response to RBCT. Previous studies reported that age could affect peripheral tissue perfusion. One study showed that SBF measured on the dorsum of the hand was 30% lower in the elderly (i.e., around 70 years) than in younger (i.e., around 20 years) healthy volunteers (52). Another study reported that elderly volunteers (mean age of 74.6 ± 7.6 years) had significantly longer capillary refill time (CRT) than younger volunteers (mean age of 32.2 ± 7.2 years) (53)_._ However, in the multivariate analysis, low SBF_BT_ remained independently predictive of a positive response, regardless of age. However, other factors did not significantly impact tissue response to RBCT. For example, storage time was not associated with an increase in SBF, despite alterations in RBCs with storage duration that might result in the release of vasodilators, a reduction in RBCs deformability, altered adhesiveness and aggregability, and the release of free Hb, which could alter microvascular flow (54). Our findings are consistent with previous reports (12, 28, 55). Also, sepsis was not independently associated with skin blood flow response to RBCT, although significant modifications in RBCs rheology were observed in septic patients (56). These findings may suggest that critical illness itself, rather than sepsis alone, could directly alter tissue perfusion and result in variable responses to different therapeutic interventions.

Another important finding in our study is that a significant reduction in the SBF was observed after RBCT in an important proportion of patients (i.e., “non-responder” group). Other clinical studies have also reported a decrease in the microvascular sublingual perfusion and tissue oxygenation when an RBCT was administered to patients who already have normal baseline tissue perfusion (12, 28). One could argue that the microcirculation is impaired by the specific rheological characteristics of the transfused RBCs. Friedlander et al. yielded that RBCT improved RBCs deformability in septic patients, probably by replacing rigidified, endogenous RBCs with more functional, or less dysfunctional exogenous RBCs (57). However, RBCT may be deleterious when performed in patients with stable microcirculatory conditions, preserved deformability, vasoreactivity, and/or tissue perfusion, probably because of an increased aggregability (13, 43). Additionally, because the flow in a capillary is proportional to the driving pressure, increments in CVP may affect the capillary perfusion pressure. Vellinga et al. reported an association between elevated CVP (>12 mmHg) and a reduction in microcirculatory blood flow in patients with sepsis (58). Other studies also reported impaired microvascular blood flow for mild increases in venous pressure in humans (59, 60). Although no significant changes in CVP were observed after RBCT in our population, we cannot exclude the possibility that increments in CVP in some of these patients could have decreased the driving pressure thereby reducing microcirculatory perfusion. However, it is also possible that this would reflect a normal physiological response of the microcirculation (i.e., vasoconstriction) in response to a supranormal oxygen delivery state.

Our study has several limitations. First, it was a single-center study, which limits its external validity. Second, during the intervention period, some interventions other than RBCT were ongoing (i.e., antibiotics, and intravenous fluids), and we cannot rule out the possibility that these changes may have influenced the final results, but they were a minority around 3.4%. Third, we did not collect other potential variables of interest, such as cardiac output, as it was not monitored in all our patients. This analysis would be informative to detect some incoherence between the microcirculatory and global systemic variables, as previously reported (12, 13, 45, 61–63). Fourth, although it would be of clinical interest to include measurements like the capillary refill time (CRT) or the mottling score, these data were not available. However, recent data confirmed the correlation between CRT and SBF measurements in critically ill patients (64). Fifth, the SLD technology itself has some limitations. SBF represents the average of the blood flow in arterioles, venules, and capillaries in the measured volume of tissue, and the relative contributions of arterial, venous, and capillary blood flow within the measured volume of tissue cannot be determined. Sixth, we cannot extrapolate skin measurements to other organs, and further studies are needed to clarify if the responses obtained at the skin microcirculatory level are the reflection of what happens in other microvascular networks.

In conclusion, the present study shows that RBCT results in variable tissue perfusion changes during critical illness. Altered skin tissue perfusion at baseline had a higher probability to improve after RBCT. Skin tissue perfusion monitoring might help to individualize transfusion strategies in this setting.

## Data availability statement

Due to ethical restrictions, the datasets used and/or analyzed during the current study are available from the corresponding author upon reasonable request. Requests to access the datasets should be directed to EC, elaine_meduece@yahoo.com.br.

## Ethics statement

The studies involving humans were approved by Erasme Hospital Ethical Committee. The studies were conducted in accordance with the local legislation and institutional requirements. The participants provided their written informed consent to participate in this study.

## Author contributions

EC and FT conceived the study. EC, PB, and DO selected the population and collected the data. EC and FT conducted the statistical analysis and wrote the first draft of the article. PB, DO, J-LV, and JC revised the text for intellectual content. All authors contributed to the article and approved the submitted version.

## References

[1] Corwin HL Gettinger A Pearl RG Fink MP Levy MM Abraham E. The CRIT study: Anemia and blood transfusion in the critically ill—current clinical practice in the United States*. Crit Care Med. (2004) 32:39–52. doi: 10.1097/01.CCM.0000104112.34142.79, PMID: 14707558

[2] Vincent JL Baron J-F Reinhart K Gattinoni L Thijs L Webb A. Anemia and blood transfusion in critically ill patients. JAMA. (2002) 288:1499–507. doi: 10.1001/jama.288.12.1499 12243637

[3] Cavalcante Dos Santos E Bakos P Vincent JL. How have red blood transfusion practices changed in critically ill patients? A comparison of the ICON and ABC studies conducted 13 years apart. Transfusion. (2020a) 60:2801–6. doi: 10.1111/trf.16048, PMID: 32888222

[4] Vincent JL Jaschinski U Wittebole X Lefrant JY Jakob SM Almekhlafi GA. Worldwide audit of blood transfusion practice in critically ill patients. Crit Care. (2018) 22:102. doi: 10.1186/s13054-018-2018-9, PMID: 29673409 PMC5909204

[5] Hébert PC Wells G Blajchman MA Marshall J Martin C Pagliarello G. A multicenter, randomized, controlled clinical trial of transfusion requirements in critical care. Transfusion requirements in critical care Investigators, Canadian critical care trials group. N Engl J Med. (1999) 340:409–17. doi: 10.1056/NEJM199902113400601 9971864

[6] Cecconi M De Backer D Antonelli M Beale R Bakker J Hofer C. Consensus on circulatory shock and hemodynamic monitoring. Task force of the European Society of Intensive Care Medicine. Intensive Care Med. (2014) 40:1795–815. doi: 10.1007/s00134-014-3525-z, PMID: 25392034 PMC4239778

[7] Juffermans NP Aubron C Duranteau J Vlaar APJ Kor DJ Muszynski JA. Transfusion in the mechanically ventilated patient. Intensive Care Med. (2020) 46:2450–7. doi: 10.1007/s00134-020-06303-z, PMID: 33180167 PMC7658306

[8] Richardson TQ Guyton AC. Effects of polycythemia and anemia on cardiac output and other circulatory factors. Am J Phys. (1959) 197:1167–70. doi: 10.1152/ajplegacy.1959.197.6.1167

[9] Vincent JL De Backer D. Circulatory shock. N Engl J Med. (2013) 369:1726–34. doi: 10.1056/NEJMra1208943 24171518

[10] Vlaar AP Wolthuis EK Hofstra JJ Roelofs JJ Boon L Schultz MJ. Mechanical ventilation aggravates transfusion-related acute lung injury induced by MHC-I class antibodies. Intensive Care Med. (2010) 36:879–87. doi: 10.1007/s00134-010-1802-z, PMID: 20221752

[11] Mueller MM Van Remoortel H Meybohm P Aranko K Aubron C Burger R. Patient blood management: recommendations from the 2018 Frankfurt consensus conference. JAMA. (2019) 321:983–97. doi: 10.1001/jama.2019.0554 30860564

[12] Sakr Y Chierego M Piagnerelli M Verdant C Dubois MJ Koch M. Microvascular response to red blood cell transfusion in patients with severe sepsis. Crit Care Med. (2007) 35:1639–44. doi: 10.1097/01.CCM.0000269936.73788.32, PMID: 17522571

[13] Scheuzger J Zehnder A Meier V Yeginsoy D Flukiger J Siegemund M. Sublingual microcirculation does not reflect red blood cell transfusion thresholds in the intensive care unit-a prospective observational study in the intensive care unit. Crit Care. (2020) 24:18. doi: 10.1186/s13054-020-2728-7, PMID: 31952555 PMC6969438

[14] Bateman RM Sharpe MD Ellis CG. Bench-to-bedside review: microvascular dysfunction in sepsis--hemodynamics, oxygen transport, and nitric oxide. Crit Care. (2003) 7:359–73. doi: 10.1186/cc2353, PMID: 12974969 PMC270719

[15] Mallat J Rahman N Hamed F Hernandez G Fischer MO. Pathophysiology, mechanisms, and managements of tissue hypoxia. Anaesth Crit Care Pain Med. (2022) 41:101087. doi: 10.1016/j.accpm.2022.101087, PMID: 35462083

[16] Siam J Kadan M Flaishon R BarneA O. Blood flow versus hematocrit in optimization of oxygen transfer to tissue during fluid resuscitation. Cardiovasc Eng Technol. (2015) 6:474–84. doi: 10.1007/s13239-015-0237-7, PMID: 26577480

[17] Dietrich KA Conrad SA Hebert CA Levy GL Romero MD. Cardiovascular and metabolic response to red blood cell transfusion in critically ill volume-resuscitated nonsurgical patients. Crit Care Med. (1990) 18:940–4. doi: 10.1097/00003246-199009000-00007, PMID: 2394117

[18] Fenwick JC Dodek PM Ronco JJ Phang PT Wiggs B Russel JA. Increased concentration of plasma lactate predicts pathological dependence of oxygen consumption on oxygen delivery in patients with adult respiratory distress syndrome. J Crit Care. (1990) 5:81–6. doi: 10.1016/0883-9441(90)90052-B

[19] Gilbert EM Haupt MT Mandanas RY Huaringa AJ Carlson RW. The effect of fluid loading, blood transfusion, and catecholamine infusion on oxygen delivery and consumption in patients with sepsis. Am Rev Respir Dis. (1986) 134:873–8. doi: 10.1164/arrd.1986.134.5.873, PMID: 3777684

[20] Lorente JA Landín L Pablo RD Renes E Rodríguez-Díaz R Liste D. Effects of blood transfusion on oxygen transport variables in severe sepsis. Crit Care Med. (1993) 21:1312–8. doi: 10.1097/00003246-199309000-00013, PMID: 8370294

[21] Cavalcante Dos Santos E Orbegozo D Mongkolpun W Galfo V Nan W Gouvea Bogossian E. Systematic review and Meta-analysis of effects of transfusion on hemodynamic and oxygenation variables. Crit Care Med. (2020b) 48:241–8. doi: 10.1097/CCM.0000000000004115, PMID: 31939794

[22] Ait-Oufella H Bige N Boelle PY Pichereau C Alves M Bertinchamp R. Capillary refill time exploration during septic shock. Intensive Care Med. (2014) 40:958–64. doi: 10.1007/s00134-014-3326-4, PMID: 24811942

[23] Ait-Oufella H Lemoinne S Boelle PY Galbois A Baudel JL Lemant J. Mottling score predicts survival in septic shock. Intensive Care Med. (2011) 37:801–7. doi: 10.1007/s00134-011-2163-y, PMID: 21373821

[24] Beerthuizen GI Goris RJ Kreuzer FJ. Skeletal muscle Po2 during imminent shock. Arch Emerg Med. (1989) 6:172–82. doi: 10.1136/emj.6.3.172, PMID: 2675881 PMC1285602

[25] Hernandez G Pedreros C Veas E Bruhn A Romero C Rovegno M. Evolution of peripheral vs metabolic perfusion parameters during septic shock resuscitation. A clinical-physiologic study. J Crit Care. (2012) 27:283–8. doi: 10.1016/j.jcrc.2011.05.024, PMID: 21798706

[26] Lima A Jansen TC Van Bommel J Ince C Bakker J. The prognostic value of the subjective assessment of peripheral perfusion in critically ill patients. Crit Care Med. (2009) 37:934–8. doi: 10.1097/CCM.0b013e31819869db, PMID: 19237899

[27] Mongkolpun W Orbegozo D Cordeiro CPR Franco CJCS Vincent J-L Creteur J. Alterations in skin blood flow at the fingertip are related to mortality in patients with circulatory shock. Crit Care Med. (2020) 48:443–50. doi: 10.1097/CCM.0000000000004177, PMID: 32205589

[28] Creteur J Neves AP Vincent JL. Near-infrared spectroscopy technique to evaluate the effects of red blood cell transfusion on tissue oxygenation. Crit Care. (2009) 13:S11. doi: 10.1186/cc8009, PMID: 19951383 PMC2786113

[29] Gouvea Bogossian E Rass V Lindner A Iaquaniello C Miroz JP Cavalcante Dos Santos E. Factors associated with brain tissue oxygenation changes after RBC transfusion in acute brain injury patients. Crit Care Med. (2022) 50:e539–47. doi: 10.1097/CCM.0000000000005460, PMID: 35132018

[30] Creteur J Carollo T Soldati G Buchele G De Backer D Vincent JL. The prognostic value of muscle StO2 in septic patients. Intensive Care Med. (2007) 33:1549–56. doi: 10.1007/s00134-007-0739-3, PMID: 17572876

[31] Gómez H Torres A Polanco P Kim HK Zenker S Puyana JC. Use of non-invasive NIRS during a vascular occlusion test to assess dynamic tissue O(2) saturation response. Intensive Care Med. (2008) 34:1600–7. doi: 10.1007/s00134-008-1145-1, PMID: 18523754

[32] Orbegozo Cortes D Puflea F De Backer D Creteur J Vincent JL. Near infrared spectroscopy (NIRS) to assess the effects of local ischemic preconditioning in the muscle of healthy volunteers and critically ill patients. Microvasc Res. (2015) 102:25–32. doi: 10.1016/j.mvr.2015.08.002, PMID: 26265192

[33] Rose JC Neill TA Hemphill JC 3rd. Continuous monitoring of the microcirculation in neurocritical care: an update on brain tissue oxygenation. Curr Opin Crit Care. (2006) 12:97–102. doi: 10.1097/01.ccx.0000216574.26686.e9, PMID: 16543783

[34] Orbegozo D Mongkolpun W Stringari G Markou N Creteur J Vincent JL. Skin microcirculatory reactivity assessed using a thermal challenge is decreased in patients with circulatory shock and associated with outcome. Ann Intensive Care. (2018) 8:60. doi: 10.1186/s13613-018-0393-7 29725778 PMC5934288

[35] Young JD Cameron EM. Dynamics of skin blood flow in human sepsis. Intensive Care Med. (1995) 21:669–74. doi: 10.1007/BF01711546, PMID: 8522672

[36] Vongsavan N Matthews B. Some aspects of the use of laser Doppler flow meters for recording tissue blood flow. Exp Physiol. (1993) 78:1–14. doi: 10.1113/expphysiol.1993.sp003664, PMID: 8448007

[37] Knaus WA Draper EA Wagner DP Zimmerman JE. APACHE II: a severity of disease classification system. Crit Care Med. (1985) 13:818–29. doi: 10.1097/00003246-198510000-00009 3928249

[38] Vincent JL Moreno R Takala J Willatts S De Mendonça A Bruining H. The SOFA (Sepsis-related organ failure assessment) score to describe organ dysfunction/failure. On behalf of the working group on Sepsis-related problems of the European Society of Intensive Care Medicine. Intensive Care Med. (1996) 22:707–10. doi: 10.1007/BF01709751, PMID: 8844239

[39] Cullis JO. Diagnosis and management of anaemia of chronic disease: current status. Br J Haematol. (2011) 154:289–300. doi: 10.1111/j.1365-2141.2011.08741.x 21615381

[40] Fogagnolo A Taccone FS Vincent JL Benetto G Cavalcante E Marangoni E. Using arterial-venous oxygen difference to guide red blood cell transfusion strategy. Crit Care. (2020) 24:160. doi: 10.1186/s13054-020-2827-5, PMID: 32312299 PMC7171832

[41] Sakr Y Vincent JL. Should red cell transfusion be individualized? Yes. Intensive Care Med. (2015) 41:1973–6. doi: 10.1007/s00134-015-3950-7, PMID: 26149304

[42] Weinberg JA Maclennan PA Vandromme-Cusick MJ Angotti JM Magnotti LJ Kerby JD. Microvascular response to red blood cell transfusion in trauma patients. Shock. (2012) 37:276–81. doi: 10.1097/SHK.0b013e318241b739, PMID: 22344313 PMC3952237

[43] Sadaka F Aggu-Sher R Krause K O'brien J Armbrecht ES Taylor RW. The effect of red blood cell transfusion on tissue oxygenation and microcirculation in severe septic patients. Ann Intensive Care. (2011) 1:46. doi: 10.1186/2110-5820-1-46, PMID: 22067279 PMC3256106

[44] Ayhan B Yuruk K Koene S Sahin A Ince C Aypar U. The effects of non-leukoreduced red blood cell transfusions on microcirculation in mixed surgical patients. Transfus Apher Sci. (2013) 49:212–22. doi: 10.1016/j.transci.2013.01.016, PMID: 23402838

[45] Tanaka S Escudier E Hamada S Harrois A Leblanc PE Vicaut E. Effect of RBC transfusion on sublingual microcirculation in hemorrhagic shock patients: a pilot study. Crit Care Med. (2017) 45:e154–60. doi: 10.1097/CCM.0000000000002064, PMID: 27635767

[46] Yuruk K Almac E Bezemer R Goedhart P De Mol B Ince C. Blood transfusions recruit the microcirculation during cardiac surgery. Transfusion. (2011) 51:961–7. doi: 10.1111/j.1537-2995.2010.02971.x, PMID: 21133930

[47] Mongkolpun W Bakos P Vincent JL Creteur J. Monitoring skin blood flow to rapidly identify alterations in tissue perfusion during fluid removal using continuous veno-venous hemofiltration in patients with circulatory shock. Ann Intensive Care. (2021) 11:59. doi: 10.1186/s13613-021-00847-z, PMID: 33855645 PMC8046875

[48] Mongkolpun W Gardette M Orbegozo D Vincent JL Creteur J. An increase in skin blood flow induced by fluid challenge is associated with an increase in oxygen consumption in patients with circulatory shock. J Crit Care. (2022) 69:153984. doi: 10.1016/j.jcrc.2022.01.001, PMID: 35078101

[49] Charkoudian N. Mechanisms and modifiers of reflex induced cutaneous vasodilation and vasoconstriction in humans. J Appl Physiol. (2010) 1985:1221–8. doi: 10.1152/japplphysiol.00298.2010 PMC2963327 20448028

[50] Cracowski JL Roustit M. Human skin microcirculation. Compr Physiol. (2020) 10:1105–54. doi: 10.1002/cphy.c190008, PMID: 32941681

[51] Walløe L. Arterio-venous anastomoses in the human skin and their role in temperature control. Temperature (Austin). (2016) 3:92–103. 27227081 10.1080/23328940.2015.1088502 PMC4861183

[52] Tsuchida Y. Age-related changes in skin blood flow at four anatomic sites of the body in males studied by xenon-133. Plast Reconstr Surg. (1990) 85:556–61. doi: 10.1097/00006534-199004000-00010, PMID: 2315395

[53] Schriger DL Baraff L. Defining normal capillary refill: variation with age, sex, and temperature. Ann Emerg Med. (1988) 17:932–5. doi: 10.1016/S0196-0644(88)80675-9, PMID: 3415066

[54] Ho J Sibbald WJ Chin-Yee IH. Effects of storage on efficacy of red cell transfusion: when is it ot safe? Crit Care Med. (2003) 31:S687–97. doi: 10.1097/01.CCM.0000099349.17094.A3 14724467

[55] Raat NJ Verhoeven AJ Mik EG Gouwerok CW Verhaar R Goedhart PT. The effect of storage time of human red cells on intestinal microcirculatory oxygenation in a rat isovolemic exchange model. Crit Care Med. (2005) 33:39–45. doi: 10.1097/01.ccm.0000150655.75519.02, PMID: 15644646

[56] Piagnerelli M Zouaoui Boudjeltia K Brohee D Vereerstraeten A Piro P Vincent JL. Assessment of erythrocyte shape by flow cytometry techniques. J Clin Pathol. (2007) 60:549–54. doi: 10.1136/jcp.2006.037523, PMID: 16775118 PMC1994557

[57] Friedlander MH Simon R Machiedo GW. The relationship of packed cell transfusion to red blood cell deformability in systemic inflammatory response syndrome patients. Shock. (1998) 9:84–8. doi: 10.1097/00024382-199802000-00002, PMID: 9488251

[58] Vellinga NA Ince C Boerma EC. Elevated central venous pressure is associated with impairment of microcirculatory blood flow in sepsis: a hypothesis generating post hoc analysis. BMC Anesthesiol. (2013) 13:17. doi: 10.1186/1471-2253-13-17 23919272 PMC3750825

[59] Christ F Dellian M Goetz AE Gamble J Messmer K. Changes in subcutaneous interstitial fluid pressure, tissue oxygenation, and skin red cell flux during venous congestion plethysmography in men. Microcirculation. (1997a) 4:75–81. doi: 10.3109/10739689709148319 9110285

[60] Christ F Gamble J Baschnegger H Gartside IB. Relationship between venous pressure and tissue volume during venous congestion plethysmography in man. J Physiol. (1997b) 503:463–7. doi: 10.1111/j.1469-7793.1997.463bh.x 9306287 PMC1159877

[61] De Backer D Creteur J Dubois MJ Sakr Y Koch M Verdant C. The effects of dobutamine on microcirculatory alterations in patients with septic shock are independent of its systemic effects. Crit Care Med. (2006) 34:403–8. doi: 10.1097/01.CCM.0000198107.61493.5A, PMID: 16424721

[62] Dubin A Pozo MO Casabella CA Pálizas F Jr Murias G Moseinco MC. Increasing arterial blood pressure with norepinephrine does not improve microcirculatory blood flow: a prospective study. Crit Care. (2009) 13:R92. doi: 10.1186/cc7922, PMID: 19534818 PMC2717464

[63] Ospina-Tascon G Neves AP Occhipinti G Donadello K Büchele G Simion D. Effects of fluids on microvascular perfusion in patients with severe sepsis. Intensive Care Med. (2010) 36:949–55. doi: 10.1007/s00134-010-1843-3, PMID: 20221744

[64] Contreras R Hernández G Valenzuela ED González C Ulloa R Soto D. Exploring the relationship between capillary refill time, skin blood flow and microcirculatory reactivity during early resuscitation of patients with septic shock: a pilot study. J Clin Monit Comput. (2023) 37:839–45. doi: 10.1007/s10877-022-00946-7 36495360

