# Supplementary material for: Transfusion increased skin blood flow when initially low in volume-resuscitated patients without acute bleeding
Source: Front Med (Lausanne). 2023 Oct 4;10:1218462. doi: 10.3389/fmed.2023.1218462 (PMC10582983; doi:10.3389/fmed.2023.1218462)
Supplement: Supplementary file 1 [file Data_Sheet_1.docx]

3Supplementary Material

Transfusion Increased Skin Blood Flow When Initially Low In Volume-Resuscitated Patients Without Acute Bleeding

Elaine Cavalcante dos Santos, MD^1*^, Péter Bakos MD^1^, Diego Orbegozo MD^1^, Jacques Creteur, MD, PhD^1^, Jean-Louis Vincent, MD, PhD^1^, Fabio Silvio Taccone, MD, PhD^1^

*** Correspondence:** Elaine Cavalcante dos Santos: elaine_meduece@yahoo.com.br

# Supplementary Figures and Tables

## Supplementary Figures

195 non bleeding patients receiving

252 transfusions were screened

Excluded (n=26 transfusions from 20 non-bleeding patients)

- Severe agitation (n=6)
- Refused consent (n=2)
- Start ultrafiltration at the moment of RBCT (n=5)
- Receipt of fluids (n=3)
- Patients who left ICU for imaging tests(n=3)
- Interruption of transfusion due to adverse effects (n=1)
- Procedures (ET, tracheostomy) (n=2)
- Patient with multidrug resistant bacteria (n=1)
- End of life procedure (n=1)
- Acute bleeding (n=1)
- Skin lesions (n=1)

175 non bleeding patients receiving

226 transfusions during ICU stay were included in the study

175 non bleeding patients receiving the first 175 transfusions were inlcuded in the final analysis for the primary and secondary outcomes

175 non bleeding patients receiving 226 transfusions were inlcuded in the sensitivity analysis

**Supplementary Figure 1.** Flow diagram of transfusion selection. ICU, intensive care unit; ET, endotracheal intubation;


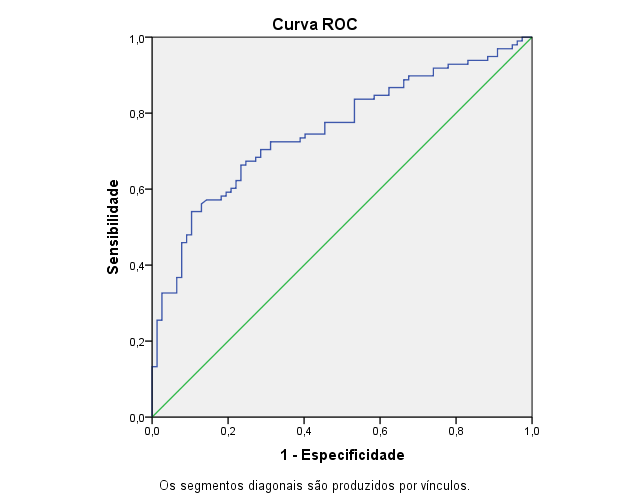


**Sensitivity**

**1- Specificity**

**Supplementary Figure 2.** Areas under the curve (AUC) for skin blood flow at basal temperature ( SBF_BT_) to identify an increase in skin blood flow at basal temperature (SBF_BT_) ≥ 20% after RBCT (Responders). The ROC curve showed that the cut-off 73.0PU for skin blood flow at basal temperature ( SBF_BT_) baseline has 71.4% sensibility and 70.4% specificity to identify responders (AUC=0.755; p<0.001).


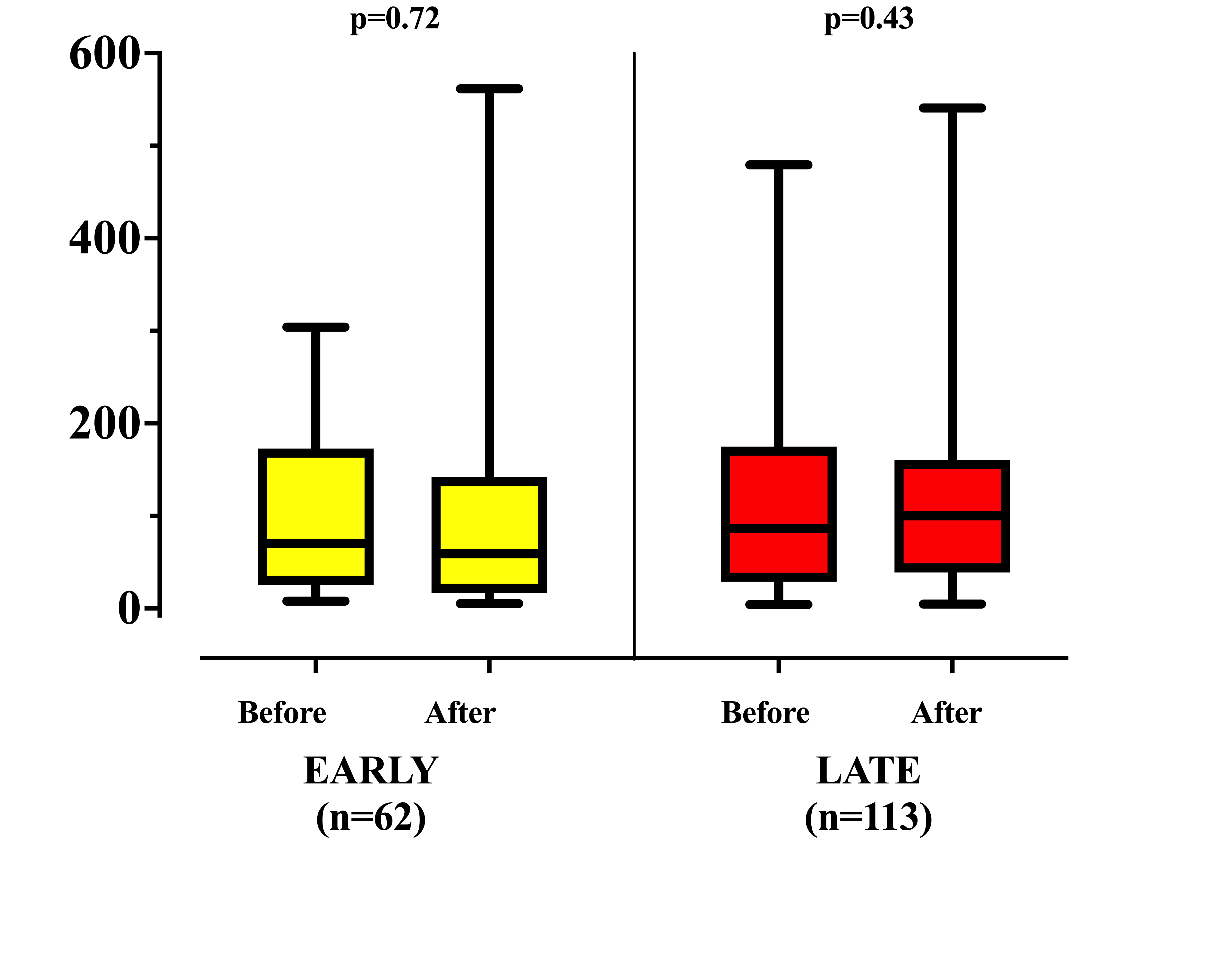


**Supplementary Figure 3.** Changes in skin blood flow at basal temperature (SBF_BT_) before and after RBCT according to the timing of transfusion after ICU admission. *p* values indicate differences between before and after RBCT in each subgroup.

**
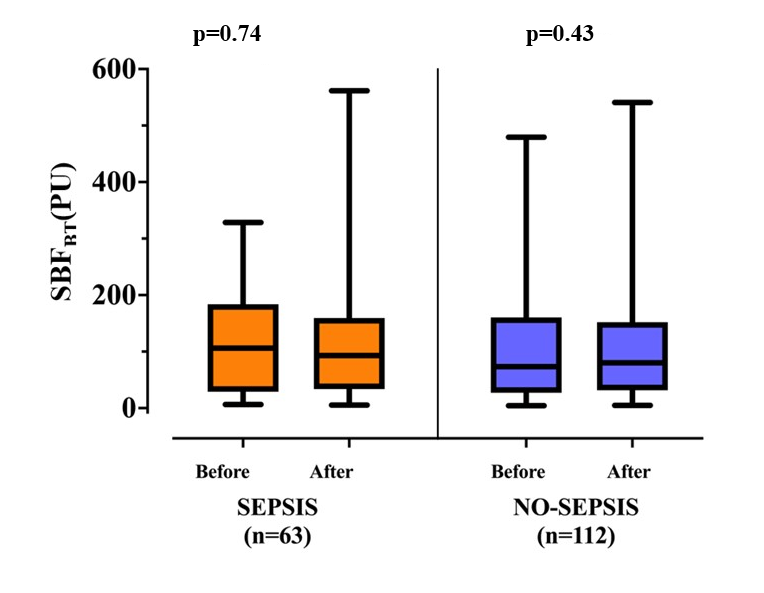
**

**Supplementary Figure 4.** Changes in skin blood flow at basal temperature (SBF_BT_) before and after RBCT according to the presence of sepsis. *p* values indicate differences between before and after RBCT in each subgroup.

**1.2 Supplementary Tables**

**Supplemental Table 1.** Multivariable analysis to identify baseline predictors of significant skin blood flow at basal temperature (SBF_BT_) increase (i.e. ≥20% from baseline value) after RBCT

|  |  | **95%CI** | |  |
| --- | --- | --- | --- | --- |
| **Variables** | **Odds ratio** | **Lower** | **Upper** | ***p* value** |
| *Acute Neurologic Disease* | 0.199 | 0.77 | 0.509 | 0.001 |
| *Age* | 1.030 | 1.002 | 1.059 | 0.038 |
| *Pre-transfusional SBF_BT_* | 0.986 | 0.982 | 0.991 | <0.001 |

Note: Data are reported as odds ratio (OR) and 95% confidence intervals (CIs).

Abbreviations: SBF_BT_, Skin blood flow at basal temperature before transfusion.

**Supplemental Table 2.** Mixed linear model to identify baseline variables independently associated with a significant skin blood flow at basal temperature (SBF_BT_) increase (i.e. ≥20% from baseline value) after RBCT

|  |  | **95%CI** | |  |
| --- | --- | --- | --- | --- |
| **Variables** | **Odds ratio** | **Lower** | **Upper** | ***p* value** |
| *Acute Neurological disease* | 0.298 | 0.136 | 0.650 | 0.003 |
| *Age* | 1.032 | 1.006 | 1.058 | 0.014 |
| *SBF_BT_* | 0.987 | 0.983 | 0.991 | <0.001 |

Note: Data are reported as odds ratio (OR) and 95% confidence intervals (CIs).

Abbreviations: SBF_BT_, Skin blood flow at basal temperature before transfusion.

**Supplemental Table 3.** Clinical characteristics and pre-transfusion skin blood flow according to the timing of red blood cell transfusion

|  | Early  (n=62) | Late  (n=113) | *p*-value |
| --- | --- | --- | --- |
| Age, years | 62.8±13.3 | 62.2±15.3 | 0.79 |
| Male Gender, n (%) | 43.0(69.4) | 68(60.2) | 0.23 |
| APACHE II score on admission | 20.0±6.7 | 22.5±7.3 | <0.01 |
| SOFA score on admission | 9.0±2.9 | 9.7±3.1 | 0.06 |
| Hemoglobin on admission, g/dL | 9.1±1.8 | 10.6±2.4 | <0.01 |
| Hematocrit on admission(%) | 27.3±5.7 | 31.7±7.1 | <0.01 |
| Admission Type, n(%) |  |  |  |
| *Surgical* | 36.0(58.1) | 36.0(31.9) | <0.01 |
| *Medical* | 22.0(35.5) | 67.0(59.3) |  |
| *Trauma* | 4.0(6.5) | 10.0(8.8) |  |
| Comorbidity, n(%) |  |  |  |
| *Arterial Hypertension* | 37.0(59.7) | 44.0(38.9) | <0.01 |
| *Diabetes* | 15.0(24.2) | 26.0(23.0) | 0.86 |
| *Coronary Arterial Disease* | 17.0(27.4) | 27.0(23.9) | 0.61 |
| *Chronic Renal Disease* | 16.0(25.8) | 26.0(23.0) | 0.68 |
| *COPD* | 8.0(12.9) | 12.0(10.6) | 0.65 |
| *Liver Cirrhosis* | 7.0(11.3) | 14.0(12.4) | 0.83 |
| *Peripheral Artery Disease* | 9.0(14.5) | 13.0(11.5) | 0.57 |
| *History of Smoking* | 10.0(16.1) | 16.0(14.2) | 0.73 |
| *Immunosuppression* | 20.0(32.3) | 23.0(20.4) | 0.08 |
| *No-Metastatic Solid Cancer* | 5.0(8.1) | 12.0(10.6) | 0.59 |
| *Metastatic Solid Cancer* | 3.0(4.8) | 1.0(0.9) | 0.09 |
| *Hematological Cancer* | 4.0(6.5) | 6.0(5.3) | 0.76 |
| *Chronic Anemia* | 22.0(35.5) | 42.0(37.2) | 0.83 |
| Reasons for Admission, n(%) |  |  |  |
| *Sepsis/Septic shock* | 16.0(25.8) | 32.0(28.3) | <0.01 |
| *Respiratory failure* | 3.0(4.8) | 5.0(4.4) |  |
| *Hypovolemic shock* | 20.0(32.3) | 6.0(5.3) |  |
| *Cardiogenic shock* | 8.0(12.9) | 23.0(20.4) |  |
| *Trauma* | 1.0(1.6) | 4.0(3.5) |  |
| *Acute neurological disease* | 7.0(11.3) | 33.0(29.2) |  |
| *Others* | 7.0(11.3) | 10.0(8.8) |  |
| RBCT characteristics |  |  |  |
| *Volume, ml* | 267.1±18.2 | 266.5±16.6 | 0.82 |
| *Age, days* | 22.0±8.4 | 24.0±7.7 | 0.05 |
| Characteristics of measurements |  |  |  |
| *Time in ICU before the study, days* | 1.0(0-2.0) | 8.0(3.0-42.0) | <0.01 |
| *Interference, n (%)* | 1.0(1.6) | 5.0(4.4) | 0.43 |
| *SBF Responder, n (%)* | 31.0(50.0) | 46.0(40.7) | 0.24 |
| *Fluid administration before study inclusion (L/day)* | 2.00±2.60 | 1.00±1.10 | <0.01 |
| Characteristics on the study day, n (%) |  |  |  |
| *Mechanical Ventilation* | 12.0(19.4) | 67.0(59.3) | <0.01 |
| *Sedation* | 9.0(14.5) | 36.0(31.9) | 0.01 |
| *Vasopressors* | 40.0(64.5) | 71.0(62.8) | 0.99 |
| During ICU stay, n (%) |  |  |  |
| *28-day mortality* | 5.0(8.1) | 24.0(21.2) | 0.03 |
| *SOFA Responder* | 42.0(67.7) | 61.0(54.0) | 0.08 |
| Baseline Data |  |  |  |
| *SOFA score on study day* | 8.2±3.2 | 8.6±3.1 | 0.41 |
| *Hemoglobin, g/dL* | 7.4±0.8 | 7.5±0.7 | 0.87 |
| *Hematocrit, %* | 18.1±2.8 | 19.7±2.7 | <0.01 |
| *RDW, %* | 16.0(12.0-28.0) | 16.0(13.0-26.0) | 0.07 |
| *Body temperature, °C* | 36.9±0.8 | 37.1±0.8 | 0.23 |
| *Mean arterial pressure, mmHg* | 77.2±10.8 | 81.5±14.3 | 0.03 |
| *Heart rate, bpm* | 92.3±16.7 | 91.1±17.4 | 0.66 |
| *Central Venous Pressure, mmHg* | 7.7±4.7 | 10.5±5.6 | <0.01 |
| *Noradrenaline dose, mcg/kg*min* | 0.14(0.01-0.90) | 0.15(0.01-4.00) | 0.39 |
| *Lactate concentration, mmol/L* | 1.4(0.3-5.6) | 1.1(0.4-5.1) | <0.01 |
| *Arterial Oxygen Saturation, %* | 98.0(85.0-100.0) | 98.0(90.0-100.0) | 0.92 |
| *Venous Oxygen Saturation, %* | 63.5±10.3 | 66.2±11.5 | 0.15 |
| *Urinary output (ml/Kg/h)* | 1.1(0.1-13.6) | 1.5(0.1-30.0) | 0.10 |
| *Finger Temperature baseline, °C* | 29.9±3.2 | 30.7±3.1 | 0.11 |
| *SBF_BT_, PU* | 70.5(7.9-304.1) | 86.5(4.3-479.4) | 0.43 |
| *SBF_37_, PU* | 110.8(10.1-444.6) | 108.5(7.7-493.2) | 0.78 |
| *∆SBF/∆T, PU/°C* | 4.2(-16.4-22.4) | 2.1(-21.9-32.9) | <0.01 |

Note: Data are reported as mean (SD), median (IQRs), or count (%).

Abbreviations: APACHE II, Acute Physiologic Assessment and Chronic Health Evaluation Scoring System II; SOFA, Sequential Organ Failure Assessment; COPD, Chronic Obstructive Pulmonary Disease; ICU, Intensive Care Unit; RDW, red blood cell distribution width; SBF_BT_, skin blood flow at basal temperature; SBF_37_, skin blood flow at 37°C; ∆SBF/∆T, (SBF_37_- SBF_BT_/T37°C-T at baseline); PU, perfusion units.

Supplemental Table 4. Comparison between pre and post-RBCT variables according to SBF baseline response to RBCT in early vs. late RBCT

|  | **Early (n=62)** | | |
| --- | --- | --- | --- |
|  | **Baseline** | **Day-1** | ***p*-value** |
| *SOFA score on study day* | 8.2±3.2 | 6.6±3.6 | <0.01 |
| *RDW* | 16.0(12.0-28.0) | 16.0(13.0-29.0) | 0.01 |
|  | **Baseline** | **1-hour** | ***p*-value** |
| *Hemoglobin, g/dL* | 7.4±0.8 | 8.7±0.9 | <0.01 |
| *Hematocrit, %* | 18.1±2.8 | 21.9±3.6 | <0.01 |
| *Body temperature, °C* | 36.9±0.8 | 36.9±0.8 | 0.84 |
| *Mean arterial pressure, mmHg* | 77.2±10.8 | 79.4±11.8 | 0.12 |
| *Heart rate, bpm* | 92.3±16.7 | 91.4±15.8 | 0.61 |
| *Central Venous Pressure, mmHg* | 7.7±4.7 | 7.5±4.7 | 0.51 |
| *Noradrenaline dose, mcg/kg/min* | 0.14(0.01-0.90) | 0.14(0.00-0.90) | 0.35 |
| *Lactate concentration, mmol/L* | 1.4(0.3-5.6) | 1.7(0.3-5.4) | 0.57 |
| *Arterial Oxygen Saturation, %* | 98.0(85.0-100.0) | 98.0(90.0-100.0) | 0.85 |
| *Venous Oxygen Saturation, %* | 63.5±10.3 | 66.6±9.0 | <0.01 |
| *Urinary output (ml/Kg/h)* | 1.1(0.1-13.6) | 1.8(0.1-9.7) | <0.01 |
| *Finger Temperature baseline, °C* | 29.9±3.2 | 29.4±3.2 | 0.06 |
| *SBF_BT_, PU* | 70.5(7.9-304.1) | 59.2(5.4-561.6) | 0.72 |
| *SBF_37_, PU* | 110.8(10.1-444.6) | 102.4(14.1-585.3) | 0.55 |
| *∆SBF/∆T, PU/°C* | 4.2(-16.4-22.4) | 2.2(-7.7-48.2) | 0.20 |
|  | **Late (n=113)** | | |
|  | **Baseline** | **Day-1** | ***p*-value** |
| *SOFA score on study day* | 8.6±3.1 | 8.0±3.4 | <0.01 |
| *RDW* | 16.0(13.0-26.0) | 16.0(12.0-25.0) | 0.02 |
|  | **Baseline** | **1-hour** | ***p-*value** |
| *Hemoglobin, g/dL* | 7.5±0.7 | 8.7±0.8 | <0.01 |
| *Hematocrit, %* | 19.7±2.7 | 23.6±3.2 | <0.01 |
| *Body temperature, °C* | 37.1±0.8 | 37.0±0.8 | 0.41 |
| *Mean arterial pressure, mmHg* | 81.5±14.3 | 83.9±14.3 | 0.02 |
| *Heart rate, bpm* | 91.1±17.4 | 90.1±17.1 | 0.25 |
| *Central Venous Pressure, mmHg* | 10.5±5.6 | 10.2±5.6 | 0.28 |
| *Noradrenaline dose, mcg/kg/min* | 0.15(0.01-4.00) | 0.13(0.00-3.00) | 0.03 |
| *Lactate concentration, mmol/L* | 1.1(0.4-5.1) | 1.1(0.5-5.2) | 0.40 |
| *Arterial Oxygen Saturation, %* | 98.0(90.0-100.0) | 98.0(86.0-100.0) | 0.40 |
| *Venous Oxygen Saturation, %* | 66.2±11.5 | 68.3±10.5 | <0.01 |
| *Urinary output (ml/Kg/h)* | 1.5(0.1-30.0) | 2.8(0.1-35.3) | <0.01 |
| *Finger Temperature baseline, °C* | 30.7±3.1 | 30.8±3.1 | 0.65 |
| *SBF_BT_, PU* | 86.5(4.3-479.4) | 100.1(4.9-540.8) | 0.43 |
| *SBF_37_, PU* | 108.5(7.7-493.2) | 131.9(13.6-423.9) | 0.26 |
| *∆SBF/∆T, PU/°C* | 2.1(-21.9-32.9) | 1.9(-57.1-26.7) | 0.47 |

Note: Data are reported as mean (SD), or median (IQRs).

Abbreviations: SOFA, Sequential Organ Failure Assessment; RDW, red blood cell distribution width; SBF_BT_, skin blood flow at basal temperature ; SBF_37_, skin blood flow at 37°C; ∆SBF/∆T, (SBF_37_- SBF_BT_/T37°C-T at baseline); PU, perfusion units.

Supplemental Table 5. Study cohort characteristics, according to the tissue response to transfusion in patients with sepsis vs. patients without sepsis (n=175)

|  | Sepsis  (n=63) | No-sepsis  (n=112) | *p*-value |
| --- | --- | --- | --- |
| Age, years | **62.2**±16.1 | 62.6±13.7 | 0.87 |
| Male Gender, n (%) | 43.0(68.3) | 68.0(60.7) | 0.32 |
| APACHE II score on admission | 24.0±6.5 | 20.0±7.2 | <0.01 |
| SOFA score on admission | 10.3±2.6 | 8.9±3.1 | <0.01 |
| Hemoglobin on admission, g/dL | 9.4±2.5 | 10.4±2.2 | <0.01 |
| Hematocrit on admission(%) | 28.2±7.4 | 31.2±6.4 | <0.01 |
| Admission Type, n(%) |  |  |  |
| *Surgical* | 25.0(39.7) | 47.0(42.0) | 0.16 |
| *Medical* | 36.0(57.1) | 53.0(47.3) |  |
| *Trauma* | 2.0(3.2) | 12.0(10.7) |  |
| Comorbidity, n(%) |  |  |  |
| *Arterial Hypertension* | 26.0(41.3) | 55.0(49.1) | 0.32 |
| *Diabetes* | 16.0(25.4) | 25.0(22.3) | 0.65 |
| *Coronary Arterial Disease* | 10.0(15.9) | 34.0(30.4) | 0.03 |
| *Chronic Renal Disease* | 13.0(20.6) | 29.0(25.9) | 0.43 |
| *COPD* | 7.0(11.1) | 13.0(11.6) | 0.92 |
| *Liver Cirrhosis* | 11.0(17.5) | 10.0(8.9) | 0.10 |
| *Peripheral Artery Disease* | 7.0(11.1) | 15.0(13.4) | 0.66 |
| *History of Smoking* | 8.0(12.7) | 18.0(16.1) | 0.55 |
| *Immunosuppression* | 19.0(30.2) | 24.0(21.4) | 0.20 |
| *No-Metastatic Solid Cancer* | 7.0(11.1) | 10.0(8.9) | 0.64 |
| *Metastatic Solid Cancer* | 2.0(3.2) | 2.0(1.8) | 0.56 |
| *Hematological Cancer* | 6.0(9.5) | 4.0(3.6) | 0.10 |
| *Chronic Anemia* | 29.0(46.0) | 35.0(31.3) | 0.05 |
| Reasons for Admission, n(%) |  |  |  |
| *Sepsis/Septic shock* | 36.0(57.1) | 12.0(10.7) | <0.01 |
| *Respiratory failure* | 1.0(1.6) | 7.0(6.3) |  |
| *Hypovolemic shock* | 2.0(3.2) | 24.0(21.4) |  |
| *Cardiogenic shock* | 8.0(12.7) | 23.0(20.5) |  |
| *Trauma* | - | 5.0(4.5) |  |
| *Acute neurological disease* | 13.0(20.6) | 27.0(24.1) |  |
| *Others* | 3.0(4.8) | 14.0(12.5) |  |
| RBCT characteristics |  |  |  |
| *Volume, ml* | 266.0±16.8 | 267.2±17.4 | 0.64 |
| *Age, days* | 23.6±8.3 | 22.8±7.9 | 0.56 |
| Characteristics of measurements |  |  |  |
| *Time in ICU before study inclusion (days)* | 5.0(0-28.0) | 3.0(0-42.0) | 0.57 |
| *Interference, n (%)* | 4.0(6.3) | 2.0(1.8) | 0.19 |
| *SBF Responder, n (%)* | 25.0(39.7) | 52.0(46.4) | 0.39 |
| *Fluid administration before study inclusion (L/day)* | 1.20(-5.00-9.50) | 0.80(-3.10-6.90) | 0.08 |
| Characteristics on the study day, n (%) |  |  |  |
| *Mechanical Ventilation* | 30.0(47.6) | 49.0(43.8) | 0.62 |
| *Sedation* | 21.0(33.3) | 24.0(21.4) | 0.08 |
| *Vasopressors* | 45.0(71.4) | 66.0(58.9) | 0.22 |
| During ICU stay, n (%) |  |  |  |
| *28-day mortality* | 14.0(22.2) | 15.0(13.4) | 0.13 |
| *SOFA Responder* | 37.0(58.7) | 66.0(58.9) | 0.98 |
| Baseline data |  |  |  |
| *SOFA score on study day* | 9.5±2.8 | 7.9±3.1 | <0.01 |
| *Hemoglobin, g/dL* | 7.1±0.6 | 7.7±0.7 | <0.01 |
| *Hematocrit, %* | 18.3±2.7 | 19.6±2.8 | <0.01 |
| *RDW* | 17.0(13.0-28.0) | 16.0(12.0-25.0) | <0.01 |
| *Body temperature, °C* | 37.1±0.8 | 36.9±0.8 | 0.11 |
| *Mean arterial pressure, mmHg* | 77.9±8.1 | 81.1±15.4 | 0.08 |
| *Heart rate, bpm* | 97.4±19.7 | 88.2±14.6 | <0.01 |
| *Central Venous Pressure, mmHg* | 10.3±6.5 | 8.9±4.7 | 0.16 |
| *Noradrenaline dose, mcg/kg/min* | 0.15(0.01-1.18) | 0.15(0.01-4.00) | 0.68 |
| *Lactate concentration, mmol/L* | 1.2(0.4-5.6) | 1.2(0.3-4.2) | 0.19 |
| *Arterial Oxygen Saturation, %* | 98.0(84.9-100.0) | 98.3(89.8-100.0) | 0.72 |
| *Venous Oxygen Saturation, %* | 67.4±10.0 | 64.1±11.7 | 0.08 |
| *Urinary output (ml/Kg/h)* | 1.4(0.1-9.0) | 1.2(0.1-30.0) | 0.52 |
| *Finger Temperature baseline, °C* | 31.0±3.2 | 30.1±3.1 | 0.07 |
| *SBF_BT_, PU* | 106.1(6.5-328.6) | 73.3(4.3-479.4) | 0.48 |
| *SBF_37_, PU* | 125.6(7.7-444.6) | 104.9(13.4-493.2) | 0.21 |
| *∆SBF/∆T, PU/°C* | 2.8(-19.3-25.4) | 2.4(-21.9-32.9) | 0.39 |

Note: Data are reported as mean (SD), median (IQRs), or count (%).

Abbreviations: APACHE II, Acute Physiologic Assessment and Chronic Health Evaluation Scoring System II; SOFA, Sequential Organ Failure Assessment; COPD, Chronic Obstructive Pulmonary Disease; ICU, Intensive Care Unit; RDW, red blood cell distribution width; SBF_BT_, skin blood flow at basal temperature; SBF_37_, skin blood flow at 37°C; ∆SBF/∆T (SBF_37_- SBF_BT_/T37°C-T at baseline); PU, perfusion units.

**Supplemental Table 6.** Comparison between pre and post-RBCT variables according to skin blood flow (SBF) baseline response to RBCT in patients with sepsis vs. patients without sepsis

|  | **Sepsis (n=63)** | | |
| --- | --- | --- | --- |
|  | **Baseline** | **Day-1** | ***p*-value** |
| *SOFA score on study day* | 9.5±2.8 | 8.6±3.6 | <0.01 |
| *RDW* | 17.0(13.0-28.0) | 17.0(14.0-29.0) | 0.27 |
|  | **Baseline** | **1-hour** | ***p*-value** |
| *Hemoglobin, g/dL* | 7.1±0.6 | 8.5±0.8 | <0.01 |
| *Hematocrit, %* | 18.3±2.7 | 22.2±3.5 | <0.01 |
| *Body temperature, °C* | 37.1±0.8 | 37.1±0.7 | 0.13 |
| *Mean arterial pressure, mmHg* | 77.9±8.1 | 80.4±10.4 | 0.01 |
| *Heart rate, bpm* | 97.4±19.7 | 95.2±18.5 | 0.02 |
| *Central Venous Pressure, mmHg* | 10.3±6.5 | 10.3±6.2 | 0.94 |
| *Noradrenaline dose, mcg/kg/min* | 0.15(0.01-1.18) | 0.15(0.00-1.00) | 0.03 |
| *Lactate concentration, mmol/L* | 1.2(0.4-5.6) | 1.3(0.5-5.4) | 0.78 |
| *Arterial Oxygen Saturation, %* | 98.0(84.9-100.0) | 97.9(91.5-100.0) | 0.20 |
| *Venous Oxygen Saturation, %* | 67.4±10.0 | 68.1±10.1 | 0.46 |
| *Urinary output (ml/Kg/h)* | 1.4(0.1-9.0) | 2.7(0.1-7.8) | <0.01 |
| *Finger Temperature baseline, °C* | 31.0±3.2 | 30.9±3.2 | 0.81 |
| *SBF_BT_, PU* | 106.1(6.5-328.6) | 93.1(5.4-561.6) | 0.74 |
| *SBF_37_, PU* | 125.6(7.7-444.6) | 131.9(14.1-585.3) | 0.71 |
| *∆SBF/∆T, PU/°C* | 2.8(-19.3-25.4) | 2.0(-17.3-29.7) | 0.49 |
|  | **No-sepsis (n=112)** | | |
|  | Baseline | 1-hour | p-value |
| *SOFA score on study day* | 7.9±3.1 | 6.9±3.3 | <0.01 |
| *Hemoglobin, g/dL* | 7.7±0.7 | 8.8±0.8 | <0.01 |
| *Hematocrit, %* | 19.6±2.8 | 23.4±3.3 | <0.01 |
| *RDW* | 16.0(12.0-25.0) | 16.0(12.0-24.0) | <0.01 |
| *Body temperature, °C* | 36.9±0.8 | 36.9±0.8 | 0.79 |
| *Mean arterial pressure, mmHg* | 81.1±15.4 | 83.4±15.0 | 0.05 |
| *Heart rate, bpm* | 88.2±14.6 | 88.0±14.9 | 0.87 |
| *Central Venous Pressure, mmHg* | 8.9±4.7 | 8.5±4.8 | 0.14 |
| *Noradrenaline dose, mcg/kg/min* | 0.15(0.01-4.00) | 0.10(0.00-3.00) | 0.55 |
| *Lactate concentration, mmol/L* | 1.2(0.3-4.2) | 1.2(0.3-4.3) | 0.61 |
| *Arterial Oxygen Saturation, %* | 98.3(89.8-100.0) | 98.2(86.0-100.0) | 0.70 |
| *Venous Oxygen Saturation, %* | 64.1±11.7 | 67.4±10.0 | <0.01 |
| *Urinary output (ml/Kg/h)* | 1.2(0.1-30.0) | 2.4(0.1-35.3) | <0.01 |
| *Finger Temperature baseline, °C* | 30.1±3.1 | 30.0±3.1 | 0.45 |
| *SBF_BT_, PU* | 73.3(4.3-479.4) | 80.2(4.9-540.8) | 0.43 |
| *SBF_37_, PU* | 104.9(13.4-493.2) | 117.6(13.6-423.9) | 0.35 |
| *∆SBF/∆T, PU/°C* | 2.4(-21.9-32.9) | 2.1(-57.1-48.2) | 0.83 |

Note: Data are reported as mean (SD), or median (IQRs).

Abbreviations: SOFA, Sequential Organ Failure Assessment; RDW, red blood cell distribution width; SBF_BT_, skin blood flow at basal temperature; SBF_37_, skin blood flow at 37°C; ∆SBF/∆T= (SBF_37_- SBF_BT_/T37°C-T at baseline); PU= perfusion units.
